# Impact of adjustable cryogel properties on the performance of prostate cancer cells in 3D

**DOI:** 10.1186/s40064-016-2629-z

**Published:** 2016-06-27

**Authors:** A. Bäcker, B. Göppert, S. Sturm, P. Abaffy, T. Sollich, F. J. Gruhl

**Affiliations:** Karlsruhe Institute of Technology (KIT), Institute of Microstructure Technology (IMT), 76344 Eggenstein-Leopoldshafen, Germany; Karlsruhe Institute of Technology (KIT), Institute of Functional Interfaces (IFG), 76344 Eggenstein-Leopoldshafen, Germany

**Keywords:** Cryogel, Stiffness, Porosity, 3D cell culture, Prostate cancer, Androgen receptor (AR) localization

## Abstract

**Background:**

Biochemical and physical characteristics of extracellular environment play a key role in assisting cell behavior over different molecular pathways. In this study, we investigated how the presence of chemical binding sites, the pore network and the stiffness of designed scaffolds affected prostate cancer cells.

**Methods:**

A blend of poly hydroxyethyl methacrylate–alginate–gelatin scaffold was synthesized by cryogelation process using polyethyleneglycol diacrylate (PEGda) and glutaraldehyde as cross linkers. The chemical and mechanical scaffold properties were varied by concentration of gelatin and PEGda, respectively. The pore network was modified by applying different ‘freezing time’. Growth, spheroid formation and localization of androgen receptor (AR) were measured to evaluate cell response within various cryogel types.

**Results:**

Insufficient porosity in combination with a brittle nature affects cell growth negatively. Spheroid size was reduced by porosity, elasticity as well as by the absence of the cell adhesive motif composed of arginine, glycine und aspartic acid (RGD). Localization of AR indicates its activity and should be under normal culture conditions in the nucleus. But in this study, we could investigate for the first time that AR remains in the cytoplasm when AR positive prostate cancer cells are cultured in scaffolds without RGD as well as in case of an insufficient pore network (total porosity under 10 %) and a too less stiffness of around 10 kPa.

**Conclusions:**

The results indicate that for getting a reliable preclinical drug screening a three-dimensional prostate model system with appropriate biochemical and physical surrounding is needed.

## Background

Designing appropriate three-dimensional (3D) scaffolds for cell culture or tissue engineering applications is one of the challenges in modern biology. The composition and the stiffness of the microenvironment surrounding the cells have major effects on cell signaling and behavior (Gasiorowski et al. 2013). Thus, due to the point that each tissue in vivo has certain characteristics, for a given study it is crucial to evaluate a corresponding in vitro matrix (Ravi et al. 2015). In general sophisticated 3D tumor spheroids have proven particularly useful for basic cancer research, such as prostate cancer, PrCa (i.e., prostaspheres (Hedlund et al. 1999; Takagi et al. 2007) to mimic the cellular in vivo environment associated with tumor growth (Thom et al. 2014; Kunz-Schughart et al. 2004). Those cell aggregates have significant potential to advance our understanding of cancer progression (Sutherland et al. 1971). Spheroids can be studied in scaffold-free suspension (e.g., ‘hanging drops’ of medium) or in scaffold-based matrices of different origin.

Among scaffold-based models are polymeric gel matrices of various materials (natural or synthetic origin) or material combinations (Nyga et al. 2011; Fischbach et al. 2007) and purified extracellular matrix (ECM) gels (e.g., Matrigel) (Streuli et al. 1991). Nevertheless, matrigel has batch-to-batch variations due to the different amounts of natural components and a very low elasticity of only 0.5 kPa, which is outside the range of certain cancer types including prostate cancer (65–126 kPa) (Soofia et al. 2009; Wells 2008). Several groups have already reported that biophysical cues of cellular microenvironment in vitro can influence the results in 3D culture, especially spheroid size (Cheng et al. 2013). Thus, the ability to control certain properties (e.g., structure, porosity as well as mechanical stimulation) is an essential parameter for engineering proper scaffolds.

Cryogels, as a type of macroporous hydrogel, synthesized at subzero temperatures, can be applied as 3D structures of different formats like fibres, meshes or sponges and offer the aforementioned controllability (Kirsebom and Mattiasson 2011). The porosity as main result of the cryotropic gelation process appears due to the existence of the solvent crystals acting as porogens for the formation of the pores (Zhao et al. 2011). The outstanding characteristics of cryogels prompted us to look for the scaffold properties and the cellular answer regarding to it to develop a suitable microenvironment for prostate cancer study in vitro. It is well-known that the synthesis parameters for cryogels are crucial for the main characteristics. Nevertheless, it is still less known about the influence on cell culture caused by physical and chemical properties of designed scaffolds.

Thus, here we report the synthesis and characterization of a polyhydroxyethyl methacrylate–alginate–gelatin–poly(ethylene glycol) diacrylate (pHAG–PEGda) cryogel combinations and their effects on culturing the epithelial prostate cancer cell line LNCaP. The polymer combination was chosen based upon the given criteria of the applied components. Gelatin has further well-known cell-adhesive properties by containing the RGD (arginine, glycine und aspartic acid) sequence, which should improve cell attachment (Aplin et al. 1998; Yamada 1991). The synthetic and water-soluble monomer poly-hydroxyethyl methacrylate (HEMA) is also highly biocompatible and is a well-known material of contact lenses (Fornasiero et al. 2005).

The aim of the present work was to investigate the effects of single physical and chemical characteristics of 3D matrices on culturing prostate cancer cells. Various changes in synthesis protocol offer the possibility to control presence of chemical attractants, like RGD sequence by use of gelatin, establishment of pore network by freezing time and degree of elasticity by cross linker concentration independently of each other. The behavior of LNCaP cells was evaluated by analyzing proliferation rate, spheroid formation and size as well as androgen receptor (AR) localization. This study can be of high importance for the establishment of a suitable 3D platform for preclinical anti-AR drug screening due to the fact that properties of the surrounding scaffold was determined to affect AR localization.

## Methods

### Materials

Gelatin (from cold water fish skin, MW ~60,000) and alginate (sodium salt, from brown algae, low viscosity ~250 cP) as well as the cross linker polyethylene glycol diacrylate (PEGda, mol wt 700) were purchased from Sigma Aldrich (Germany). The monomer poly-hydroxyethyl methacrylate (HEMA) was obtained from Alfa Aesar (Karlsruhe, Germany). The initiators ammonium persulfate (APS) and N,N,N′,N′-tetramethylethylene diamine (TEMED) as well as the cross linker glutaraldehyde were purchased from Merck (Darmstadt, Germany). RPMI Medium 1640 ([+] l-Glutamin), Phosphate-buffered saline (PBS) and l-Glutamine (200 mM) were purchased from Life Technologies. Fetal bovine serum (FBS) and Antibiotic Antimycotic solution were purchased from Sigma Aldrich.

### Methods

#### Preparation of pHAG–PEGda cryogels

Different concentrations of poly-hydroxyethyl methacrylate (pHEMA), gelatin, alginates and poly(ethylene glycol) diacrylate (PEGda) were used to synthesize the poly-hydroxyethyl methacrylate–alginate–gelatin–poly(ethylene glycol) diacrylate (pHAG–PEGda) cryogels in water as solvent. The gels were prepared by mixing gelatin and alginate in double-distilled water. The monomer HEMA and the cross linker PEGda were added to the reaction mixture after complete dissolution of the previous polymers. The gel reaction formation was initiated by adding APS/TEMED (0.5 %/0.1 % w/v) and glutaraldehyde (25 % v/v) as gelatin crosslinker. The mixture was poured immediately into 3 ml syringe and frozen overnight (otherwise it will be mentioned in the text) at −21 °C. The polymerized cryogels were cutted into 3 mm thick disks with a diameter of 8 mm, thawed at room temperature and stored in double-distilled water at 4 °C prior to use.

#### Microstructure analysis using scanning electron microscopy (SEM)

The pore network morphology of cryogels was studied using scanning electron microscope (SEM, Zeiss 60VP). The scaffolds were dried over 2 days at room temperature to remove moisture totally before silver coating was performed using a sputter coater (EMITECH K575X). The microscope was operated at high vacuum of 3.00 kV. If cells were integrated in scaffolds after certain time of cell culture, the samples were first fixed with 2.5 % glutaraldehyde in 0.1 M phosphate buffered saline (PBS, pH 7.4) and dried at least 2 days at room temperature.

#### Visco-elastic behavior of scaffolds

Compression tests were carried out on RPMI medium saturated cryogels (disks) without cells. Uniaxial stress was given directly to the cylindrical samples using Zwick/Roell Z0.5 machine (Germany) with 50 N load cell by a constant linear compression rate of 1.0 mm/min. Here the elastic modulus of the cryogels was calculated from the linear region of the stress–strain curve at low strain regions. All measurements were made five times.

#### Porosity analysis

Mercury intrusion porosimetry (MIP) is a well-known technique used to measure pore size distribution in a porous material. The pore size distribution, pore surface area and pore volume in a cryogel samples was determined using the mercury porosimetry machine (Thermo Finnigan, Pascal 140/440) (Woerly et al. 1998; Lv et al. 2005). The samples were placed into penetrometers which are weighed in front of the experiment. The analysis is based on the principle that a non-wetting liquid (mercury) penetrates into the porous network only under pressure. To determine the porosity, it was assumed that the shape of the pores was cylindrical. The contact angle of mercury is 130°, and the surface tension of mercury is 0.485 N m^−1^. The pressure applied is inverse proportional to the inner width of the pore aperture.

The pore diameter was calculated using the Washburn equation:

$$D = - \left( {4\gamma *\cos \theta } \right)/P,$$

where P is the applied pressure, θ the contact angle and ɣ the surface tension of mercury.

The intrusion volume was recorded for pressure ranging from 0 to 480 MPa. The porosity was calculated using the equation:

$$\left[ {\frac{ds - db}{ds}} \right]*100,$$

where ds is the skeletal density of the dried gel (derived from gel volume with exclusion of the volume of the sample’s pore structure) and db is the bulk density of the dried gel (derived from the gel volume that includes the volume of the sample’s pore structure).

#### Cell culture and proliferation

Human prostate epithelial cancer cells (LNCaP) with cell suspension (2.5 × 105 cells/µl) were seeded onto sterilized pHAG-PEGda cryogels. The samples were pre-sterilized with RPMI medium supplements with 10 % FCS and 1 % Antibiotic Antimycotic Solution followed by repeated washing with sterilized PBS (pH 7.4). After 2 h of initial cell attachment, the samples were transferred to 6 well culture plates, containing medium and incubated at 37 °C in a humidified 5 % CO_2_ atmosphere. Fresh medium was replenished every 2 days. At regular time intervals the cryogels were fixed with 2.5 % glutaraldehyde for scanning electron microscopy analysis. Or in case of paraffin sections with subsequent haematoxylin and eosin (H&E) staining the samples were fixed with 3.7 % formalin. The total metabolic activity of the cells was monitored using CellTiter-Glo^®^ 3D (Promega). The CellTiter-Glo^®^ 3D Viability Assay is a homogenous method to determine the number of viable cells in 3D cell culture model systems. The assay based on quantitation of the ATP, which is a marker for the presence of metabolically active cells. In brief, the media was removed from the cell-seeded cryogels and followed by cooling down at room temperature for 30 min. The ready-to-use reagent as well as fresh medium was added with the equal volume to the samples. The measured amount of adenosine triphosphate (ATP) is proportional to the amount of DNA.

#### Haematoxylin/eosin (H&E) and immunofluorescence (IF) staining

For H&E staining, the reaped cryogels were fixed in 3.7 % formalin solution, dehydrated and embedded in paraffin. Several sections (5 µm thick) were deparaffinized. The deparaffinized sections were subsequently stained by hematoxylin which reacts like a basic dye with a purple-blue color and eosin an acidic dye that is typically reddish or pink. Hematoxylin stains acidic, or basophilic, structure including the cell nucleus. In contrast to that eosin stains basic, or acidophilic, structures including the cytoplasm, cell walls, and extracellular fibers (Fischbach et al. 2007). After finishing the staining, the sections were covered with a cover glass (to make the preparation permanent).

For IF, the deparaffinized sections were washed three times PBS (pH 7.4). The non-specific binding sites were blocked with block buffer (including 10 % goat serum, 0.1 % Tween 20) for 1 h and the sections were then incubated for 120 min at room temperature in mouse anti-Androgen Receptor primary antibody (Dianova DLN-09477) at 1:25 in humid environment. After rinsing with PBS (three times), the sections were incubated for an additional 1 h in goat-anti-mouse Cy3 at 1:200 in humid environment. The sections were counterstained with DAPI at 1:5000 and examined under an inverted fluorescence microscope (Leica DM5500).

#### Statistical analysis

All quantitative measurements were performed on at least triplicates. All values are expressed as means ± standard deviations (SD). One-way ANOVA with post hoc Tukey tests were used to compare treatment groups in the quantitative measurements and p < 0.05 was used to assess statistical significance.

## Results

### Physical characterization of different pHAG-PEGda cryogels

**Fig. 1:**
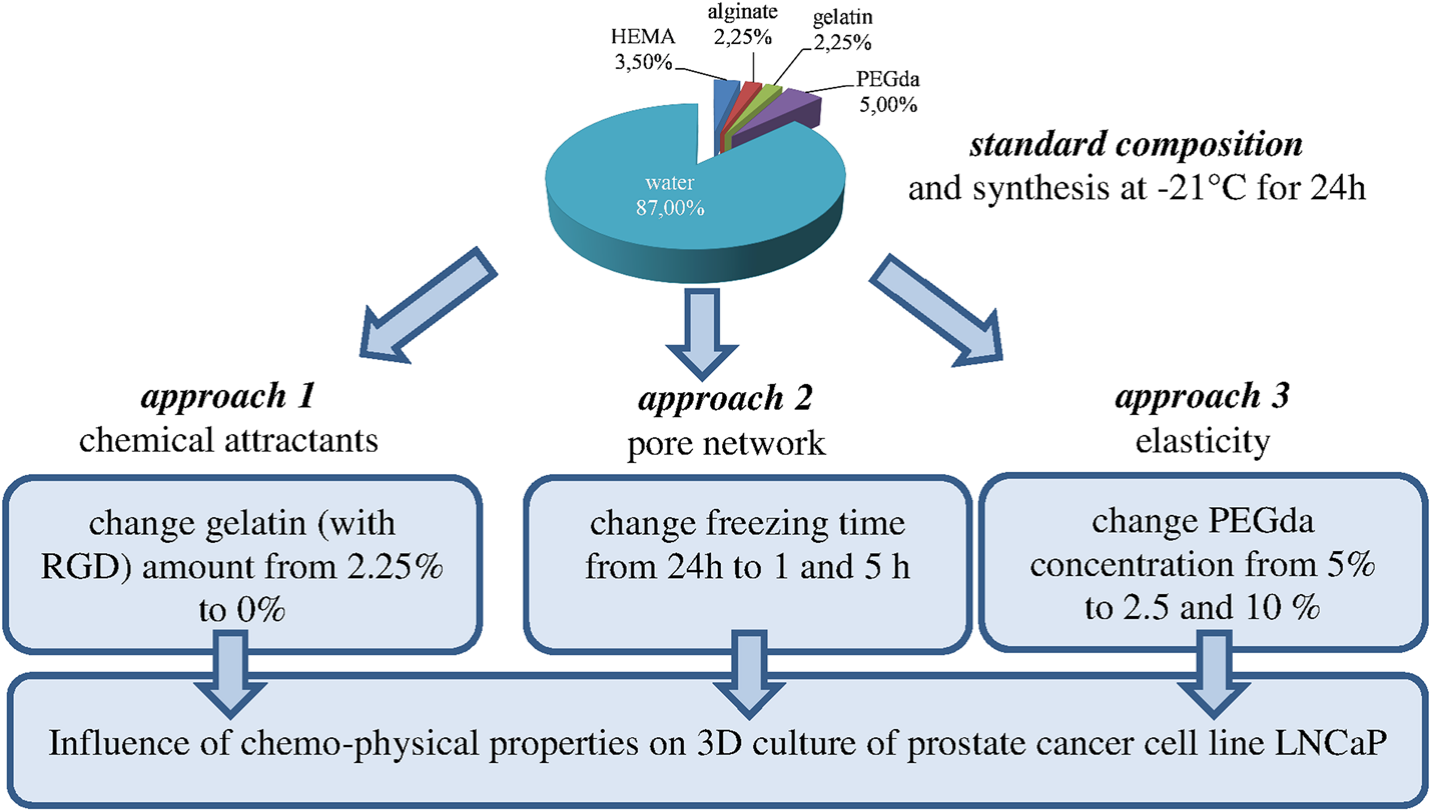
Schematic illustration of experimental approaches. *Standard* composition (percentage of components given in the cake diagram) synthesized at −21 °C for 24 h serves as reference, which was changed chemically by leaving gelatin (*approach 1*) as well as physically by varying freezing time (*approach 2*) and concentration of cross linker (*approach 3*)

**Fig. 2:**
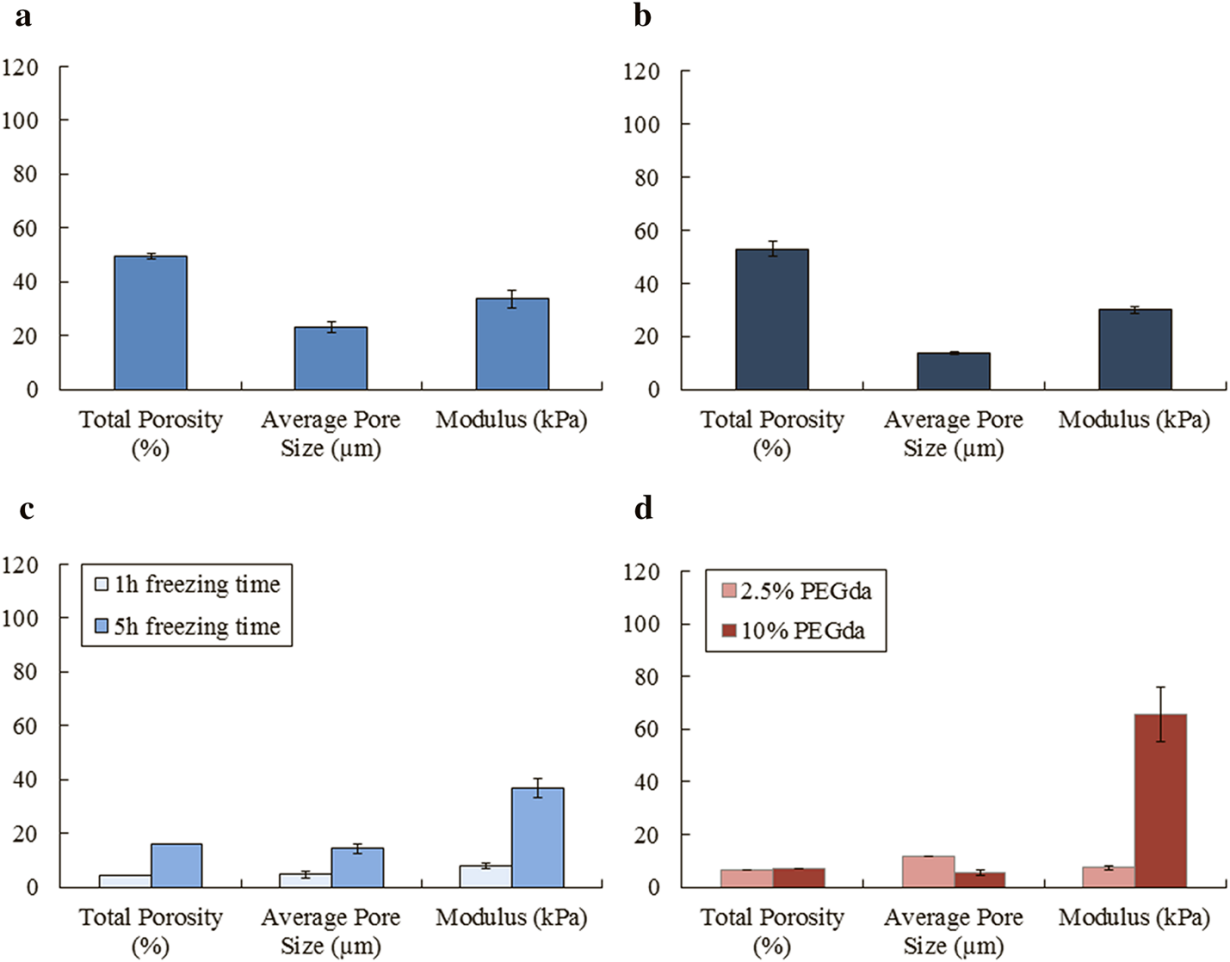
Physical characteristics (total porosity, average pore size and elasticity) of **a** standard, **b** approach 1 (without gelatin), **c** approach 2 (changes freezing time) and **d** approach 3 (changed PEGda concentration cryogels. Porosity was measured by mercury intrusion porosimetry and elasticity by compressive strength test. The experiments were done in triplicate. *Error bars* represent standard faults

Cryogels were synthesized at −21 °C and concentrations of components applied as mentioned in Fig. 1. Under standard synthesis conditions a cryogel with 50 % total porosity, an average pore size of 20 µm and a stiffness of 38 kPa was generated (Fig. 2a). The absence of gelatin (approach 1) has no influence on porosity and elasticity, respectively and only a small reduction of the average pore size (Fig. 2b). Varying freezing-time (approach 2) has a considerable effect on the architectural features of the cryogel. A cryogelation time of only 1 h, leads to less mechanical stability and a brittle nature, which was confirmed by an E modulus of only 10 kPa (Fig. 2c). However, elasticity of the standard cryogel (around 40 kPa) is already reached at 5 h and thus further elongation of freezing time remains without any effect. In comparison to that the effect of freezing time on porosity as well as average pore size is clearly detectable by a stepwise increase of these parameters according to elongating the freezing time from 1 h over 5 h up to the standard of 24 h (Fig. 2a, c). It was observed that lower amount of PEGda concentration (2.5 %) compared to standard protocol (5 %) leads to bigger pore sizes as well as thinner pore walls (pictures not shown). These results could be confirmed by measuring the average pore size, which are decreased (Fig. 2d). Whereas total porosity is influenced in both cases and leads to a significantly decrease down to 10 %. Doubling the amount of PEGda to 10 % leads to an increase of elasticity from 40 to 65 kPa and halving it to 2.5 % decreases elasticity from 40 to 10 kPa (Fig. 2d).

### Influence of scaffold properties on proliferation and spheroid formation of epithelial prostate cancer cell line LNCaP

**Fig. 3:**
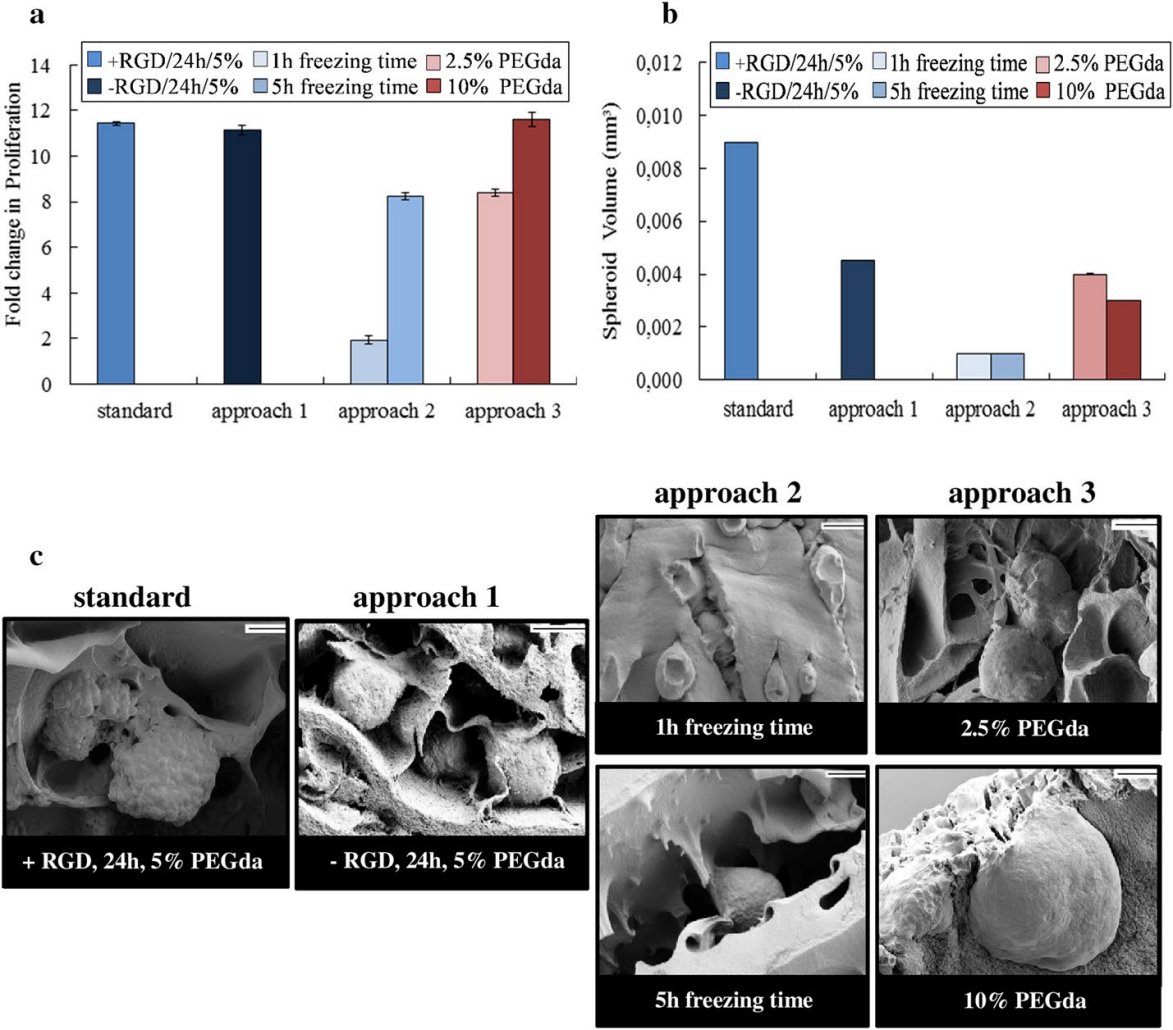
Growth and spheroid formation of LNCaP cells. **a** Total metabolic activity analysis of epithelial prostate cancer cells LNCaP seeded in different cryogel scaffolds after 21 days. The viability and proliferation of LNCaP cells was evaluated by applying CellTiter-Glo^®^ 3D Viability assay. Fold change in proliferation was calculated by normalizing the cell number at day 21 to that at day 0. **b** Calculated volume of LNCaP spheroids after 21 days cell culture. The spheroid width and length was determined by the software of the microscope (AxioVision LE64) of the corresponding HE-slices, which results in the spheroid volume. The experiments were done in triplicate. Error bars represent standard faults. **c** SEM images of LNCaP spheroids on different cryogel matrices after 21 days of culture at ×35 magnification. *Scale bar* 200 µm

As we could demonstrate, the lack of gelatin (approach 1) has no negative effect on the physical properties (Fig. 2b). Nevertheless, gelatin contains the RGD sequence, which is essential for cell adhesion. Interestingly the effect of this chemical attractants on cell growth is not as much as expected (Fig. 3a), but the absence of gelatin leads to a minimization of the spheroid size from 0.009 mm^2^ (within the standard cryogel) to 0.004 mm^2^ (Fig. 3b). However, spheroid shape looks similar—round and regular—independently of RGD presence (Fig. 3c).

After 3 weeks an obvious reduction in proliferation was obtained, which suggests proliferation stop or even dying of cells (Fig. 3a, approach 2, 1 h). The impact of higher elasticity is already visible with cryogels frozen for 5 h, which shows only small differences to proliferation rate of cryogels frozen for 24 h (Fig. 3a, approach 2, 5 h). A stiffer cryogel (Fig. 3, approach 3, 10 % PEGda) improves proliferation at day 21. To investigate, if our results are dependent on the cell type we measured additionally the proliferation rate of the prostate cancer cell line PC3 according to the influence of RGD presence and elasticity. The presence (standard) or absence of RGD (approach 1) has no significant influence on the proliferation rate of LNCaP as well as PC3 (Table 1). In contrast to that stiffer cryogels (approach 3, 10 % PEGda) increase growth of PC3 cells only. Whereas soft matrices (approach 3, 2.5 % PEGda) reduce cell growth in both cases.

**Table 1:** Fold change in proliferation of various prostate cancer cell lines in cryogels

Cryogel type	LNCaP	PC3
Standard (+RGD; 5 % PEGda)	4.14 ± 0.195	4.63 ± 0.058
Approach 1 (−RGD; 5 % PEGda)	5.21 ± 0.074	4.41 ± 0.005
Approach 3 (+RGD; 2.5 % PEGda)	3.17 ± 0.123	2.67 ± 0.003
Approach 3 (+RGD; 10 % PEGda)	4.74 ± 0.170	7.92 ± 0.112

n = 3, mean ± SEM; proliferation measured at day 7

Furthermore, it was observed that support of nutrient transport by suitable porosity and pore size combined with stable scaffold given by elasticity, leads not only to the highest cell proliferation, but also to well- and round-shaped spheroids with a volume of nearly 0.01 mm^3^ (Fig. 3b, c, standard). Spheroid shape is independent of chemical attractants like RGD sequences, which should improve cell adhesion to the matrix, however decreasing spheroid size to 0.004 mm^2^ (Fig. 3b, c, approach1).

The principle low growth rate and even decreased proliferation of cells within cryogels, which was freezed for only 1 h (Fig. 3a, approach 2), becomes coherent by analyzing spheroid volume and shape. In all spheroids is a necrotic core visible (Fig. 3c, approach 2). Interestingly the spheroid size of 0.001 mm^3^ is comparable with the volume of spheroids grown in cryogels of approach 2, which was freezed for 5 h (Fig. 3b). Nevertheless, proliferation rate was positively affected by increased porosity caused by longer freezing time of 5 h (Fig. 3a, approach2). For cell proliferation the differences in elasticity seems to have no influence within the first 14 days of LNCaP cell culture (Table 1), whereas a long-term cultivation needs mechanical strength and stiffer material to increase cell growth (Fig. 3a). Interestingly spheroid size decrease in case of higher stiffness (Fig. 3b, approach 3, 10 % PEGda) more than with low elasticity (Fig. 3b, approach 3, 2.5 % PEGda) compared to spheroid size of standard cryogels.

### Influence of scaffold properties on AR localization of epithelial prostate cancer cell line LNCaP

**Fig. 4:**
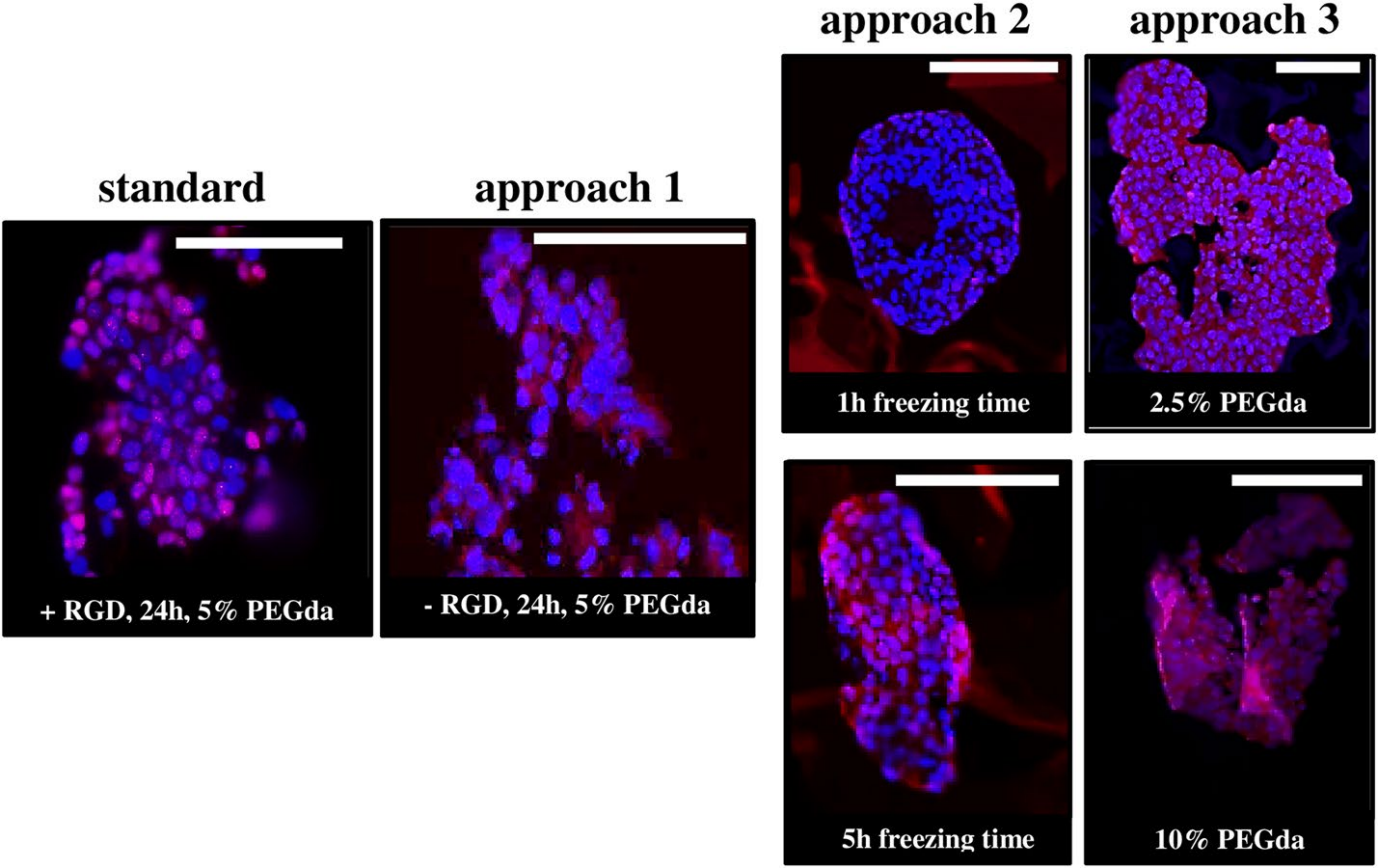
Localization of androgen receptor (AR) in LNCaP cells seeded in various cryogel types after 21 days. Immune fluorescence staining against AR (*red*) and counterstaining with DAPI (*blue*) of the corresponding paraffin Sections (10 µm) was done. *Scale bar* 200 µm

In a scaffold without gelatin, like it was given in approach 1, no RGD sequence is present. We could observe that this fact leads to a cytoplasmatic localization of AR, although hormone is still available (Fig. 4, approach 1). Furthermore, in our work we could investigate by applying approach 2 that less porosity and pore size leads also to an AR location in cytoplasm (Fig. 4, approach 2, 1 h). It seems to be that after 5 h freezing time and thus doubling the porosity as well as the average pore size AR is localized frequently in the nucleus (Fig. 4, approach 2, 5 h). A similar behavior could be observed by changing elasticity with applying approach 3. Decreasing elasticity to around 10 kPa leads to an AR localization in cytoplasm, whereas AR is represented in nucleus by increasing stiffness of matrix to nearly 60 kPa (Fig. 4, approach 3).

## Discussion

After generating a ‘standard’ cryogel and 3 variations of that according to chemical composition, pore network as well as elasticity the main physical characteristics were analyzed. The absence of gelatin (approach 1) in the material composition affects obviously none morphology nor elasticity. For cryogelation process the formation of ice crystals is the most important parameter as a template for generating the macroporous network, which can be tuned by freezing temperature and time. It is known that a freezing temperature of −10 to −21 °C leads to an optimal interconnected ice crystal system (Svec 2010). Therefore, we kept the freezing temperature stable at −21 °C and usually the optimal proposed period of 24 h. To investigate the principle influence of freezing time we focused on that in approach 2 and reduced the freezing time in two steps to 1 and 5 h, what influences mainly porosity and pore size. Whereas the cross linker concentration (approach 3) the mechanical strength and elasticity. The higher the amount of cross linker, the stiffer is the material. The fact that porosity decrease in correlation to stiffer and denser material with small pore sizes and thick pore walls (10 % PEGda), but also in correlation with very spongy and elastic material, is reasoned by collapsed pores under pressure and allows the fluid (i.e., mercury) during the measurement to be pressed through the material and enlarge by this way the pores (2.5 % PEGda). Hence, mechanical strength influences indirectly the pore network. The cryogel formation recommends that the cryogelation process depends upon the temperature of freezing and the rate of crosslinking. An increasing of initial monomer/polymer as well as cross linker concentration leads to a faster crosslinking rate at the same temperature (−21 °C). The gelation process in the polymer solution starts before the porogens (ice crystals) are formed (Kathuria et al. 2009). The faster crosslinking rate implicates formed gels with less porosity, less mechanical strength and brittle nature. The current study reveals the possibility to manipulate the cryogel properties with single parameter of the synthesis process including material composition, freezing time and amount of cross linker.

In order to investigate the effect of chemical and physical scaffold characteristics on 3D cell culturing to design an appropriate prostate model, we initially cultivated the epithelial prostate cancer cell line LNCaP within the varied cryogel matrices up to 21 days and analyzed growth, spheroid formation as well as androgen receptor (AR) localization.

Proliferation analysis showed that the lack of chemical attractants such as RGD sequence can be compensated by a suitable porosity and pore size. And vice versa, the combination of too less porosity, small average pore size and low elasticity cannot be compensated, even not by presence of RGD. Thus, incorporation of chemical attractants such as RGD might be important for cell attachment on the scaffold especially for the first days, but is ineffective and not necessary anymore, respectively, for long-term 3D culturing regarding growth rate.

We suggest therefore, that long-term culture of cells depends more on sufficient elasticity, which stands for tissue-like behavior as well as for mechanical strength and sufficient nutrient supply given by stabilization of the pore network. Nevertheless, the higher elasticity (approach 3, 10 % PEGda) did not increase cell proliferation compared to the standard cryogel. But this result could be reasoned by applying LNCaP cell line, which is relatively independent to effects of stiffness (Tilghman et al. 2010). We could confirm this hypothesis by applying another prostate cancer cell line (PC3), which is highly metastatic and derived from bone metastasis (Kaighn et al. 1979). Stiffer cryogels reduplicate the growth rate of PC3. We suggest that higher E modulus of 3D matrices stimulates growth of cells which are of metastatic origin and tend to metastasize after a certain time in culture, respectively. Furthermore, RGD presence or absence showed no effect on cell growth also in this case.

H&E staining enabled to analyze the formation and size of the tumor spheroids. The smaller the average pore size, the smaller the spheroid. Increasing cross linker concentration to design stiffer cryogels (approach 3, 10 % PEGda) affects porosity and pore size negatively by forming thicker pore walls and a denser matrix in general. Interestingly same effects could be obtained by decreasing elasticity (approach 3, 2.5 % PEGda), which is in this case due to the collapse of pores given by mechanical weakness. Therefore, it is possible to minimize spheroid size and maintaining high cell proliferation by doubling the stiffness of the surrounding scaffold.

In our study we checked the androgen receptor (AR) localization by immune fluorescent staining of LNCaP cells cultured for 21 days within the various cryogel types. Androgens play a critical role in the development and progression of PCa by controlling androgen-regulated genes (ARGs) mediated via AR, a transcription factor. Upon ligand binding the cytosolic AR is translocated into the nucleus, where it binds to the promotors of ARGs. It is well-known that AR signaling and androgen responsiveness of LNCaPs appear to be sensitive to serial passaging and different culture conditions. Indeed, we could conclude from our work that AR localization depends on RGD presence, porosity and elasticity. The fact that RGD and the interaction between tumor cells and ECM, respectively, affect expression of ARGs was already investigated by other workers (Robbins et al. 1996; Sieh et al. 2012). But as far as we know this is the first hind that porosity and elasticity of surrounding environment have a direct and independent influence on cell behavior over AR localization. It seems to be that certain porosity as well as a certain elasticity range leads to remaining of AR in the cytoplasm although hormones are present in the standard medium. Such findings confirm the strong need for a reliable model system for anti-AR drug screening, which mimic the in vivo situation as good as possible and avoid influences by the artificial surrounding as well.

## Conclusion

For designing a well-defined 3D cell culture system to study prostate cancer it is mandatory to know about the crosstalk between matrix and cells. The absence of chemical attractants (i.e., RGD) does not influence physical parameters and interestingly does not improve cell proliferation, but reduces spheroid size. The proliferation rate depends strongly on physical scaffold properties and showed a stepwise increase of cell number by improving the pore network. The effect of less elasticity on cell proliferation is comparable with those results obtained in scaffolds with an insufficient pore network. The most outstanding effect we could examine was the influence of chemical and physical properties on the localization of the androgen receptor. It is expected that AR is localized in the nucleus to activate ARGs in the presence of hormones like it is in the case of applying standard medium. But storage in the cytoplasm could be observed in scaffolds without RGD, with a low porosity and a low elasticity. This fact is not related with spheroid size or proliferation rate.

Thus, this study is a step towards a tailor-made scaffold, which provided precise and well-defined environment focused on the so important rigidity and density of matrix for prostate cancer cells. Furthermore, only small variations in synthesis already enable to guide cells dependent on research questions which shall be answered with this system. Therefore, the suggested 3D cryogel scaffold could be the basis for developing a tunable cell culture platform for cancer research and screening for anticancer drugs, especially anti-AR drugs.

## Abbreviations

3D: three-dimensional
APS: ammonium persulfate
AR: androgen receptor
ARG: androgen-regulated genes
ATP: adenosine triphosphate
ECM: extracellular matrix
FBS: fetal bovine serum
H&E: haematoxylin and eosin
HEMA: poly-hydroxyethyl methacrylate
IF: immunofluorescence
MIP: mercury intrusion porosimetry
PBS: phosphate-buffered saline
PEGda: polyethyleneglycol diacrylate
pHAG: poly hydroxyethyl methacrylate–alginate–gelatin
PrCa: prostate cancer
RGD: tripeptide amino acid sequence arginine, glycine und aspartic acid
TEMED: N,N,N′,N′-tetramethylethylene diamine
